# Improving the Stability of a Hemipelvic Prosthesis Based on Bone Mineral Density Screw Channel and Prosthesis Optimization Design

**DOI:** 10.3389/fbioe.2022.892385

**Published:** 2022-05-30

**Authors:** Rongqi Zhou, Haowen Xue, Jincheng Wang, Xiaonan Wang, Yanbing Wang, Aobo Zhang, Jiaxin Zhang, Qing Han, Xin Zhao

**Affiliations:** Department of Orthopedics, The Second Hospital of Jilin University, Changchun, China

**Keywords:** bone mineral density, topological optimization, porous structure, hemipelvic prosthesis, screw channel

## Abstract

In pelvic reconstruction surgery, the hemipelvic prosthesis can cause significant changes in stress distribution due to its high stiffness, and its solid structure is not suitable for osseointegration. The purpose of this study was to identify a novel bone mineral density screw channel and design the structure of the prosthesis so as to improve the distribution of stress, promote bone growth, and enhance the biomechanical properties of the prosthesis. The mechanical characteristics of bone mineral density screw and traditional screw were compared by finite element analysis method, and redesigned by topology optimization. The direction of the newly proposed screw channel was the posterolateral entrance of the auricular surface, ending at the contralateral sacral cape. Compared to the original group, the maximum stress of the optimized prosthesis was decreased by 24.39%, the maximum stress of the sacrum in the optimized group was decreased by 27.23%, and the average strain energy density of the sacrum in the optimized group was increased by 8.43%. On the surface of screw and connecting plate, the area with micromotion more than 28 μm is reduced by 12.17%. On the screw surface, the area with micromotion more than 28 μm is reduced by 22.9%. The newly determined screw channel and optimized prosthesis design can effectively improve the biomechanical properties of a prosthesis and the microenvironment of osseointegration. This method can provide a reference for the fixation of prostheses in clinical pelvic reconstruction.

## 1 Introduction

Primary pelvic sarcoma seriously impacts the survival and quality of life of patients. Limb-salvage surgery through prosthesis placement or biological reconstruction is more popular than traditional hemipelectomy (Muller et al., 2002). With the progress to date in artificial prostheses and surgical technology, many limb-salvage methods have been developed, such as the implantation of a modular hemipelvic endoprosthesis (Ji et al., 2010), a customized endoprosthesis (Iqbal et al., 2017), or a massive allograft (Matejovsky et al., 2006). Most of these approaches have achieved good results in follow-up (Zhou et al., 2011; Zhou et al., 2013). However, when patients experience recurrence or prosthesis instability, orthopedics doctors face significant challenges in resolving the issue (Ji et al., 2013).

During sacral pelvic reconstruction and repair, a screw–rod system (Zang et al., 2014) and cancellous bone screw (Zhao X. et al., 2018) are two common hemipelvic prosthesis–fixation methods that may be used. However, these fixation methods are often accompanied by some complications, such as neurovascular injury (Zhao Y. et al., 2018; Alkhateeb et al., 2020), screw loosening (Lu et al., 2000), and fracture (Shao et al., 2015). Previous studies have shown that patients with osteoporosis are more prone to low-energy traumatic fractures (den Teuling et al., 2017) and pedicle screw–loosening (Weiser et al., 2017). Related studies have measured and analyzed sacral bone mineral density (BMD) (Salazar et al., 2015; Thiesen et al., 2020). However, few studies to date have characterized the effect of BMD on the strength of sacral screw fixation. At present, there is no study reporting the results of finite element analysis (FEA) of sacral screw fixation and BMD. Therefore, we used FEA to verify the relationship between the difference of BMD and the strength of sacral screw fixation.

The huge mismatch in mechanical properties between the sacrum and prosthesis materials can easily lead to a stress-shielding effect (Al-Tamimi et al., 2017), and significant bone resorption may occur around the prosthesis as a result of this effect (Iolascon et al., 2010). To reduce the amount of stress shielding, structural design methods are needed to render the stress distribution between the prosthesis and bone more uniform (Liu et al., 2021). Topology optimization (TO) is one such method of structural design. It provides the best shape of the structure from the specified area under certain design considerations, such as load and boundary conditions (Park et al., 2019). Through the TO approach, the stress between the prosthesis and bone can be better distributed. In addition, based on the TO design, adding a porous structure on the surface of the screw and prosthesis can reduce the elasticity modulus of the prosthesis, providing a suitable microenvironment for bone growth and maintaining the stability of the prosthesis (Zhang et al., 2020). Therefore, this study combines TO technology with a porous structure. A porous structure can effectively reduce stiffness, so it has been widely used in orthopedics (Hu et al., 2019; Chen et al., 2020).

In this study, we aimed to measure the sacral BMD and determine the position of the screw according to the BMD distribution, then conduct FEA to verify its mechanical stability. Combined with a TO design, a porous structure was added on the surface to obtain good stability, promote bone growth, and reduce complications.

## 2 Materials and Methods

### 2.1 BMD Measurement

**FIGURE 1:**
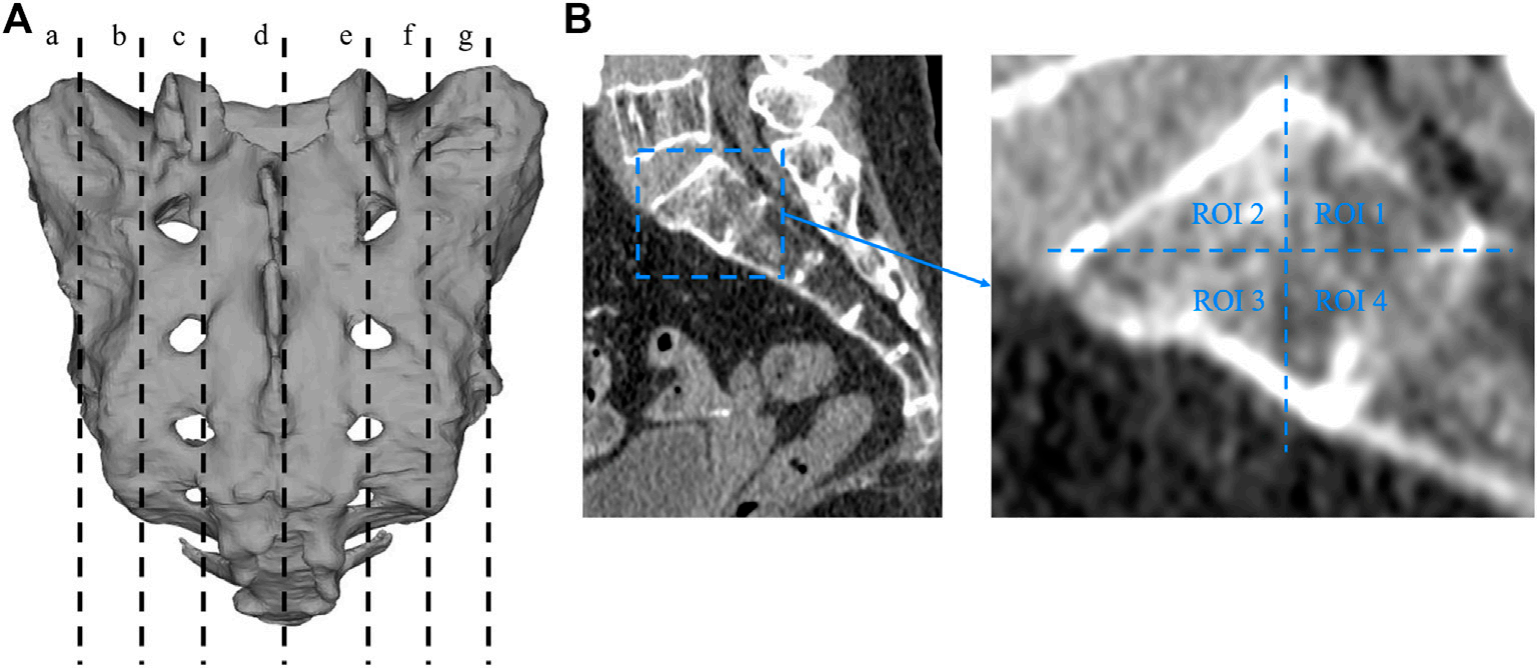
Sectional diagram of seven layers of sacral sagittal plane and the measurement method of BMD. **(A)** a: The junction of the left auricular surface and the dorsal side. b: Middle of the left sacral wing. c: Left margin of the sacral canal. d: Median sacral line. e: Right margin of the sacral canal. f: Middle of the right sacral wing. g: The right auricular surface is at the junction with the dorsal side. **(B)** The sagittal plane is divided into four regions, and the bone mineral density of each region is measured with a region-of-interest tool in the OsiriX Lite software.

Two researchers collected computed tomography (CT) data from 40 patients, including 20 men and 20 women, with an average age of 67.23 ± 4.26 years (range, 33–79 years). The CT data were imported into the medical image–processing software OsiriX Lite (Pixmeo SARL, Bernex, Switzerland), which was used to measure the BMD in the sagittal plane of the patient’s sacrum. Seven layers of sacral sagittal plane were scanned for BMD measurement, including the BMD of the sagittal sections of the bilateral auricular surface, bilateral sacral ala midline, bilateral sacral canal edge, and sacral midline. A sectional diagram of the seven layers of the sacral sagittal plane and the measurement method were shown in Figure 1. The cancellous bone region of the S1 level was divided into four regions of interest (ROIs), and the average BMD of each region was measured using an ROI tool.

### 2.2 Three-Dimensional (3D) Modeling

**FIGURE 2:**
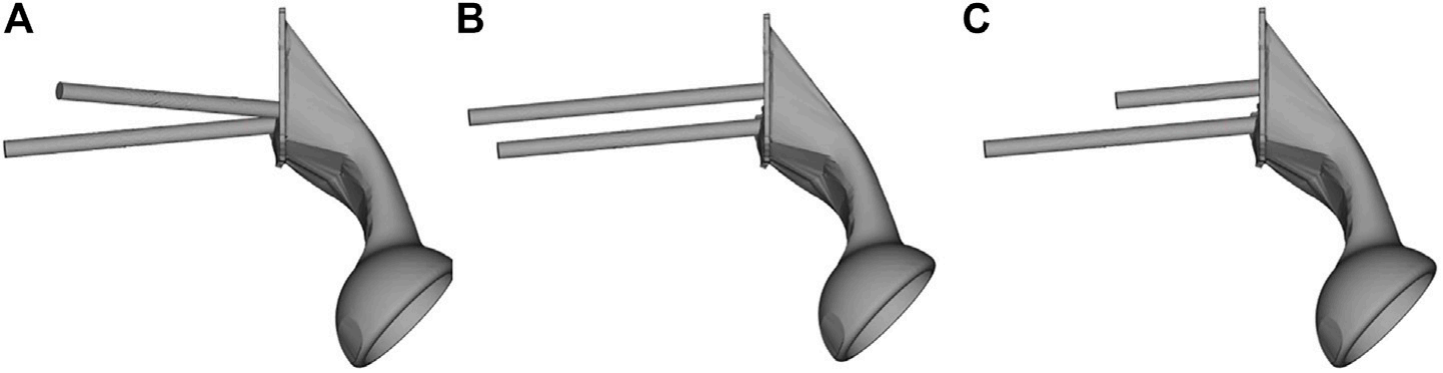
The S1 and S2 screws are tightly connected to the hemipelvic prosthesis with acetabulum through a connecting plate. The screw is replaced by a cylinder. **(A)** Bone mineral density screw. **(B)** Long screw, and **(C)** short screw.

The geometry of the sacral model was based on CT data of the pelvis of a female volunteer. Pelvic CT data were acquired using the iCT 256 scanner with an X-ray tube (Philips, Amsterdam, Netherlands) with the following parameters: current, 232 mA; voltage, 120 kV; pixel size, 0.52 mm; section thickness, 0.9 mm; and spacing between sections, 0.45 mm. The CT data were imported into the Mimics version 21.0 software program (Materialise, Leuven, Belgium), and the sacrum was reconstructed into a 3D model. The 3D model of the sacrum was then imported into Magics version 24.0 (Materialise), and its left auricular surface was intercepted. On this basis, three S1 screw channels (long screw, short screw, and BMD screw) were designed, and fixed with a long screw at the S2 level. BMD screw placement is a way to implant screws near a high BMD area. A hemipelvic prosthesis with acetabulum was designed and reconstructed using Magics version 24.0. The hemipelvic prosthesis was mainly composed of three parts: a connecting plate in contact with the auricular surface, an acetabular cup, and the connecting device present between the connecting plate and the acetabular cup. The screws were tightly connected with the prosthesis through the connecting plate and fixed on the auricular surface. These screws were simplified to cylinders for later application of FEA. The design of the three parts of the prosthesis and screws is shown in Figure 2. This study was approved by the ethics committee of the Second Hospital of Jilin University, and we obtained the informed consent of all participants.

### 2.3 FEA Model

**FIGURE 3:**
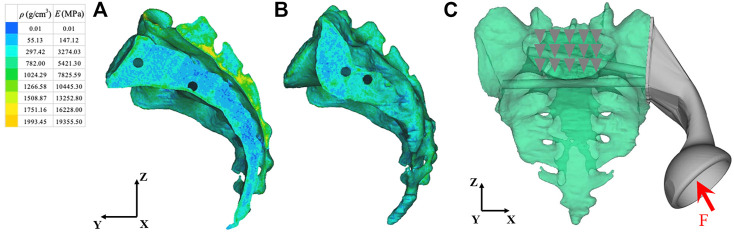
Material properties of inhomogeneous sacrum and model load and constraint. **(A)** Internal material properties of the sacrum. **(B)** External material properties of the sacrum. **(C)** A load was applied to the center of the acetabulum. The constraint was set on the upper surface of S1 vertebral body. *ρ*: Bone mineral density; E: Elastic modulus. The arrow and triangles represent the load and constraint of the FEA respectively.

We imported the 3D model of the prosthesis into the HyperMesh 2020 software program (Altair engineering, Troy, Michigan, United States). The triangle-based surface mesh in the prosthesis model is set to 1 mm using self-animation mesh technology. After generating the two-dimensional mesh, it is transformed into a 4-node linear tetrahedral element (C3D4). Using Mimics, a 3D sacral model with inhomogeneous material properties was defined according to the gray value of the CT scan. The material properties of inhomogeneous sacrum are shown in Figure 3. According to previous literature (Rho et al., 1995), the material properties of the sacrum were determined according to the following equations:

$$\text{ρ}\left(g/m^{3}\right)=47+1.122\times GV\left(HU\right)$$

$$E\left(Pa\right)=0.63\times \rho^{1.35}g/m^{3}$$

where ρ is the bone density, GV is the gray value of the bone in CT data, and E is the elastic modulus. The element size of the sacral model was set to 1 mm. In the analysis, the material was assumed to be of a linear elastic type. According to previous literature, the Poisson’s ratio of bone was set to 0.3 (Dong et al., 2018). The material properties of the finite element model were determined according to our previous research data (Liu et al., 2021). The material characteristics of various implants and sacrum were shown in Table 1.

**TABLE 1:** The material characteristics of various components.

Component	Material	Elastic modulus (MPa)	Poisson’s ratio	Element
BMD screw	Ti6Al4V	110,000	0.3	9,793
Long screw	Ti6Al4V	110,000	0.3	16,544
Short screw	Ti6Al4V	110,000	0.3	6,998
S2 screw	Ti6Al4V	110,000	0.3	12,737
Connecting plate	Ti6Al4V	110,000	0.3	22,049
Hemipelvic prosthesis	Ti6Al4V	110,000	0.3	177,820
Sacrum	Inhomogeneous bone	Inhomogeneous bone	0.3	747,607

The load conditions of the static analysis carried out in this study were the same as those used by (Bergmann et al., 2001). A load of 1948 N was applied to the center of the acetabulum, and the magnitude and direction of the force were normalized to 100%. In terms of boundary conditions, the area of the upper surface of S1 vertebral body was considered to be completely limited. The load and boundary conditions of prosthesis and sacrum are shown in Figure 3. The simulation process adopted quasi-static loading nonlinear analysis, with 20 steps of iteration until convergence, and the iterative method adopted Newton Raphson method. The stress distribution and relative micromotion were selected as the main parameters to verify the stress shielding effect and bone growth effect. The results were obtained and measured in HyperView (Altair Engineering, Troy, MI, United States). Based on the previous research data (Bartolomeu et al., 2019), the friction type of each contact surface was determined, and the contact surface was regarded as a nonlinear contact surface. The types of friction between components were shown in Table 2.

**TABLE 2:** Friction types between components.

Contact surface A	Contact surface B	Friction type
Sacrum	Porous surface	μ1 = 0.667, μ2 = 0.431
Sacrum	Solid surface	μ1 = 0.647, μ2 = 0.348
Connecting plate	Screw	Stick

Note: μ_1_ is the static friction coefficient, μ_2_ is the dynamic friction coefficient.

### 2.4 TO of Screws and Connecting Plate

According to the stress distribution of the prosthesis, the BMD screw and connecting plate were topologically optimized. TO of the connecting plate and screws was performed in HyperMesh 2020. Referring to previous literature (Chen and Shih, 2018), the minimally compliant TO under the volume fraction constraint was adopted. The optimization equation was as follows:

Objective function: to minimize (Uc).

Constraint: 0 < η_i_ < 1 (i = 1, 2, 3 … n)

$$V\leq V_{0}-V*$$

$$V=\underset{i}{\sum }\eta_{i}V_{i}$$

$$E_{i}=E\left(\eta_{i}\right)$$

$$\left\{\sigma_{i}\right\}=\left[E_{i}\right]\left\{\varepsilon_{i}\right\}$$

where U_c_ is the compliance, η_i_ represents the internal pseudodensity assigned to each finite element (i) in the optimization equation, V is the computed volume, V_0_ is the original volume, V* represents the amount of volume to be removed, V_i_ is the volume of element i, E_i_ is the elasticity tensor for each element, E represents the elasticity tensor, σ_i_ is the stress vector of element i, and ε_i_ represents the strain vector of element. η, as the density index, ranged from 0 to 1. An η value close to 0 indicates the material to be removed, and an η value close to 1 indicates the material to be retained. The program was set to reduce the volume by up to 50% and iterate 30 times at most.

Next, we input the results of TO into Magics version 21.0. The parts to be removed were obtained by performing Boolean operations between the intact model and the optimized parts obtained by TO. In this study, the newly designed prosthesis retained the same shape as the original prosthesis. The removed parts and optimized parts were designed to have a high-strength body-centered cubic structure. The removable part was designed with an optimal porosity of 70% and an aperture of 200 μm to allow for early and extensive bone ingrowth (Arabnejad et al., 2016; Bartolomeu et al., 2019). The porosity of the optimized part of the screw surface and the connecting plate was 30% and the pore diameter was 200 μm to maintain stiffness and promote proper bone growth (Chen et al., 2017; Bartolomeu et al., 2019). Then, the biomechanical changes between the original prosthesis and the optimized prosthesis were compared through FEA.

The FEA results were processed using HyperView 2020. Von Mises stress values of the sacrum and prosthesis were recorded. The prosthesis consists of screws, a connecting plate, and the hemipelvic prosthesis. The strain energy density (SED) can be used as an index of shielding stress (Ahmed et al., 2020). Inserting a relatively rigid prosthesis into the bone will lead to a reduction in the distribution of non-physiological load and bone strain around the prosthesis. The insertion of the prosthesis reduces the physiological load borne by bone, so the stress is shielded. In addition, when stress shielding occurs, the strain of the bone around the prosthesis will be reduced. Therefore, a high SED indicates low stress shielding (Zhang et al., 2020).

### 2.5 Data Analysis

Using the SPSS software (IBM Corporation, Armonk, NY, United States), the BMD data were statistically analyzed by one-way ANOVA. Paired *t*-test was used to statistically analyze the data of topology optimized prosthesis. Statistical significance was set as *p* < 0.05.

## 3 Results

### 3.1 BMD Measurement Results

In the measurement, it was found that the BMD distribution on both sides of the sacrum was symmetrical. The mean and standard deviation values of BMD were determined in Hounsfield units (Thiesen et al., 2020). The measured sacral BMD data are shown through one-way analysis of variance in Table 3 and BMD of the ROI in seven sagittal planes (mean ± standard deviation, Hu) in Table 4. In the junction between the left auricular surface and the dorsal side, the BMD in the fourth quadrant was the highest, and the average value was 182.33 ± 39.31 HU (*p* < 0.01). In the middle of the left sacral ala, the first quadrant had the highest BMD, with an average value of 103.90 ± 39.04 HU (*p* < 0.01). In the left margin of the sacral canal, the BMD in the third quadrant was the highest, and the average value was 264.51 ± 88.75 HU (*p* < 0.01). In the middle sacral line, the BMD in the second quadrant was the highest, with an average value of 240.81 ± 65.30 HU (*p* < 0.01). In the right margin of sacral canal, the BMD in the third quadrant was the highest, and the average value was 247.19 ± 67.47 HU (*p* < 0.01). In the middle of the right sacral ala, the first quadrant had the highest BMD, with an average value of 107.02 ± 39.09 HU (*p* < 0.01). Finally, at the junction of the right auricular surface and the dorsal side, the BMD in the fourth quadrant was the highest, with an average value of 187.21 ± 52.91 HU (*p* < 0.01).

**TABLE 3:** Results of one-way analysis of variance of sacral bone mineral density.

ANOVA		SS	v	MS	F	PR
A	Group	69,351.674	3	23,117.225	14.812	0.000
	Residual	243,464.596	156	1,560.670	—	—
	Total	312,816.270	159	—	—	—
B	Group	116,910.076	3	38,970.025	27.723	0.000
	Residual	219,285.470	156	1,405.676	—	—
	Total	336,195.546	159	—	—	—
C	Group	178,988.181	3	59,662.727	8.127	0.000
	Residual	1,145,263.065	156	7,341.430	—	—
	Total	1,324,251.246	159	—	—	—
D	Group	303,818.741	3	101,272.914	34.849	0.000
	Residual	453,344.150	156	2,906.052	—	—
	Total	757,162.891	159	—	—	—
E	Group	171,339.808	3	57,113.269	11.810	0.000
	Residual	754,385.772	156	4,835.806	—	—
	Total	925,725.580	159	—	—	—
F	Group	158,005.185	3	52,668.395	48.005	0.000
	Residual	171,155.495	156	1,097.151	—	—
	Total	329,160.680	159	—	—	—
G	Group	71,624.126	3	23,874.709	9.597	0.000
	Residual	388,070.197	156	2,487.629	—	—
	Total	459,694.323	159	—	—	—

**(A)** the junction of the left auricular surface and the dorsal side; **(B)** middle of the left sacral wing; **(C)** left margin of the sacral canal; **(D)** median sacral line; **(E)** right margin of the sacral canal; **(F)** middle of the right sacral wing; **(G)** the right auricular surface is at the junction with the dorsal side. *Abbreviations:* ANOVA, analysis of variance; F, F value, MS, mean square; PR, *p* value; SS, sum of squares; v, degrees of freedom.

**TABLE 4:** BMD of the region of interest in seven sagittal planes (mean ± SD, Hu).

Group (*N* = 40)	Mean ± SD			
	ROI 1	ROI 2	ROI 3	ROI 4
A	135.11 ± 41.98	133.88 ± 41.85	167.09 ± 34.40	182.33 ± 39.31
B	103.90 ± 39.04	97.49 ± 44.18	51.34 ± 31.89	42.96 ± 33.62
C	203.03 ± 79.37	241.83 ± 114.22	264.51 ± 88.75	178.25 ± 46.31
D	140.25 ± 45.63	240.81 ± 65.30	212.83 ± 54.59	143.71 ± 47.94
E	187.44 ± 68.42	239.41 ± 88.44	247.19 ± 67.47	170.86 ± 47.83
F	107.02 ± 39.09	100.91 ± 31.64	45.74 ± 31.43	37.36 ± 29.53
G	142.55 ± 42.51	134.73 ± 44.84	170.63 ± 57.73	187.21 ± 52.91

A, the junction of the left auricular surface and the dorsal side; B, middle of the left sacral wing; C, left margin of the sacral canal; D, median sacral line; E, right margin of the sacral canal; F, middle of the right sacral wing; G, the right auricular surface is at the junction with the dorsal side. *Abbreviations:* Average: average value; N, number of samples; ROI, region of interest; SD: standard deviation.

### 3.2 Von Mises Stress of the Original Prosthesis

**FIGURE 4:**
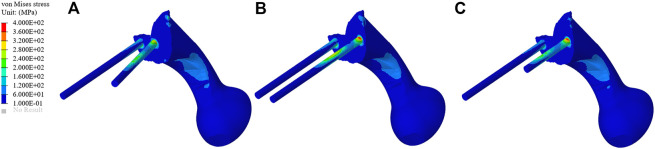
Stress distribution of the three different fixation methods. **(A–C)** The stress distribution of the bone mineral density screw, long screw, and short screw.

The stress distribution of the prosthesis varies according to the three different fixation methods. As shown in Figure 4, on the BMD screw, the stress is mainly distributed at the connection between the connecting plate and S1 screw, and no obvious stress concentration was observed. The maximum stress is 350.33 MPa, which is located at the base of the S1 screw. On the long screw, the stress is mainly distributed in the first half of the S1 screw, and an obvious stress concentration could be observed at the connection position between the S1 screw and connecting plate, with the maximum stress being 442.34 MPa. On the short screw, the stress distribution is similar to that on the long screw, and the stress concentration is located at the connection between the S1 screw and connecting plate, with the maximum stress being 446.92 MPa.

### 3.3 TO Results and von Mises Stress of the Sacrum and Prosthesis

**FIGURE 5:**
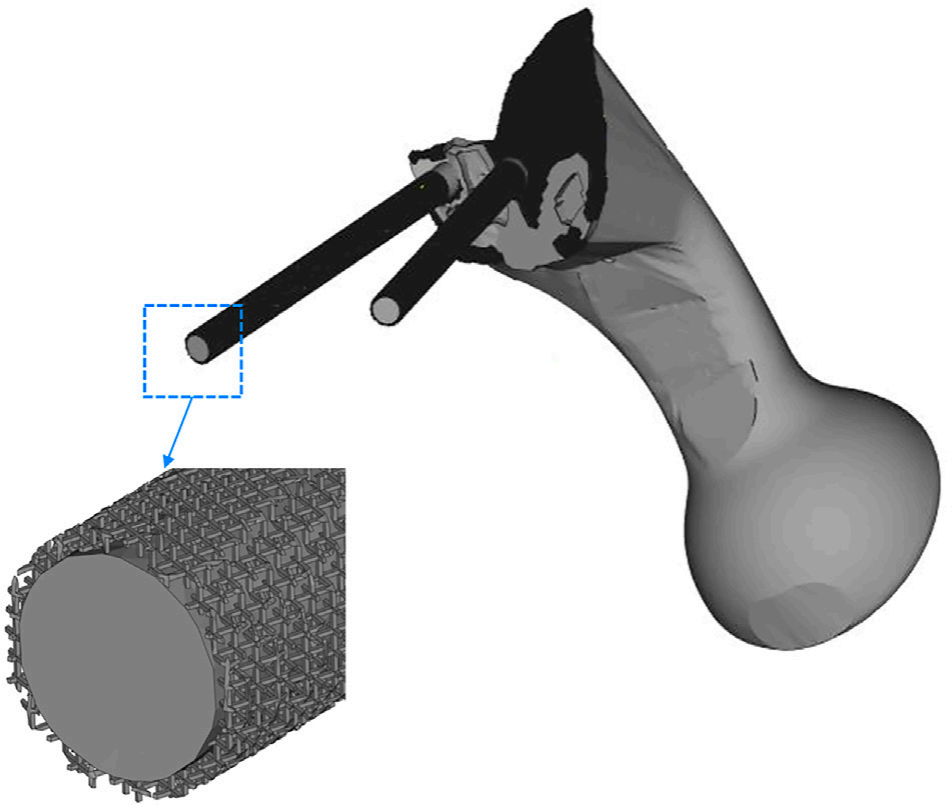
The middle two areas of the connecting plate are optimized and removed, and a porous structure with a porosity of 30% was added to the screw surface and the high-density area of the connecting plate.

The TO program was iterated six times. The algorithm mainly removed the middle two portions of the connecting plate; about 84% of the connecting plate was retained. As shown in Figure 5, the connecting plate and screws with porous structure were newly added to the surface. The porosity of the removed parts was designed to be 70%, and that of the optimized connecting plate and screws surface was designed to be 30%.

**FIGURE 6:**
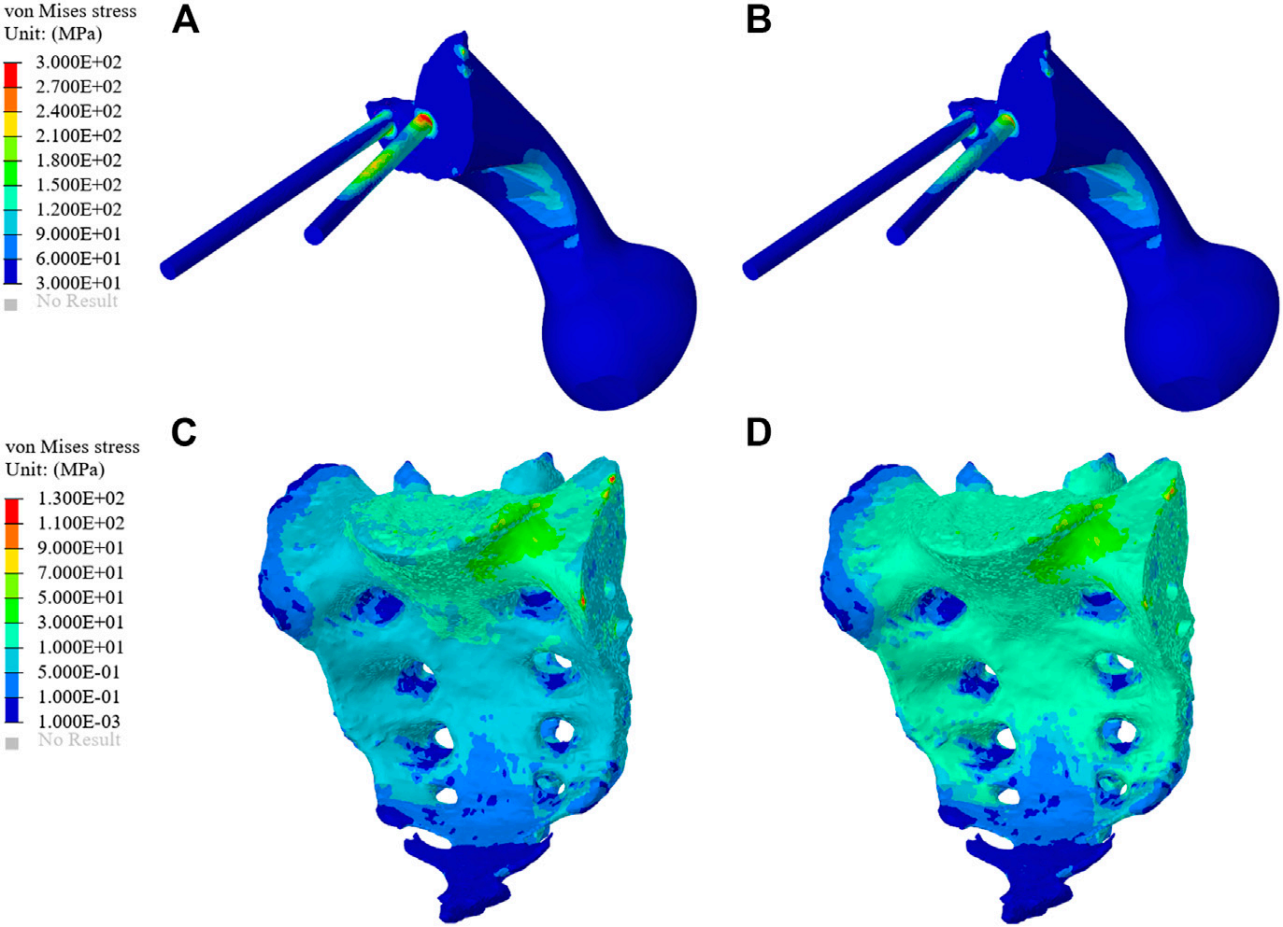
Stress comparison of the prosthesis and sacrum between the original group and the optimization group. **(A)** The stress distribution of prosthesis in the original group. **(B)** The stress distribution of prosthesis in the optimization group. **(C)** The stress distribution of the sacrum in the original group. **(D)** The stress distribution of the sacrum in the optimization group.

**FIGURE 7:**
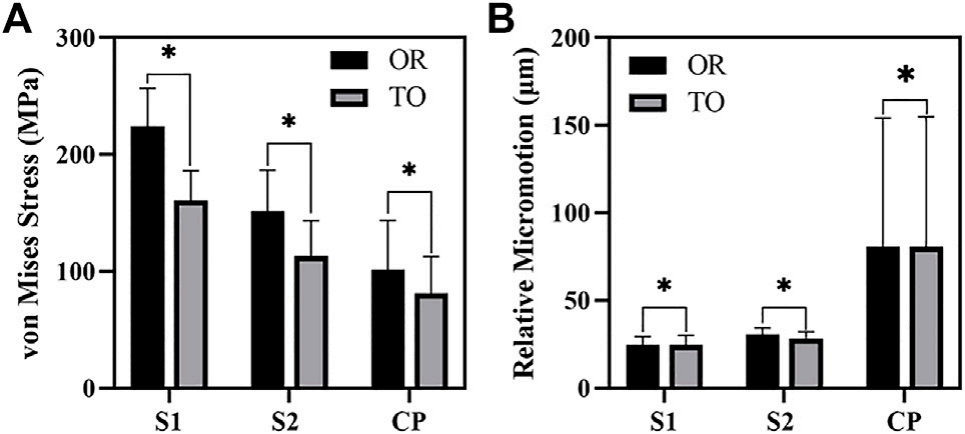
The results of von Mises Stress and relative micromotion on S1, S2 and CP. **(A)** The results of average stress. **(B)** The results of average micromotion. S1, S2 represented the S1, S2 screw, and CP represented the connecting plate. OR and TO represented original group and topological optimization group, respectively. * represented *p* < 0.05.

As shown in Figure 6, the von Mises stress values of the original group and the optimized group. The maximum stress of the original prosthesis was 350.33 MPa; after TO, however, the maximum stress of the prosthesis was reduced to 264.87 MPa, demonstrating a decrease of 24.39%. In addition, the high-stress area of the optimized prosthesis was significantly reduced. Moreover, the maximum stress of the sacrum in the original group was 183.36 MPa, while that in the optimized group was 133.43 MPa, showing a decrease of 27.23%. Figure 7A shows the average stress on S1 screw, S2 screw and connecting plate. The stress of each screw and connecting plate in the optimized group was significantly lower than that in the original group (*p* < 0.05).

### 3.4 SED of the Sacrum

**FIGURE 8:**
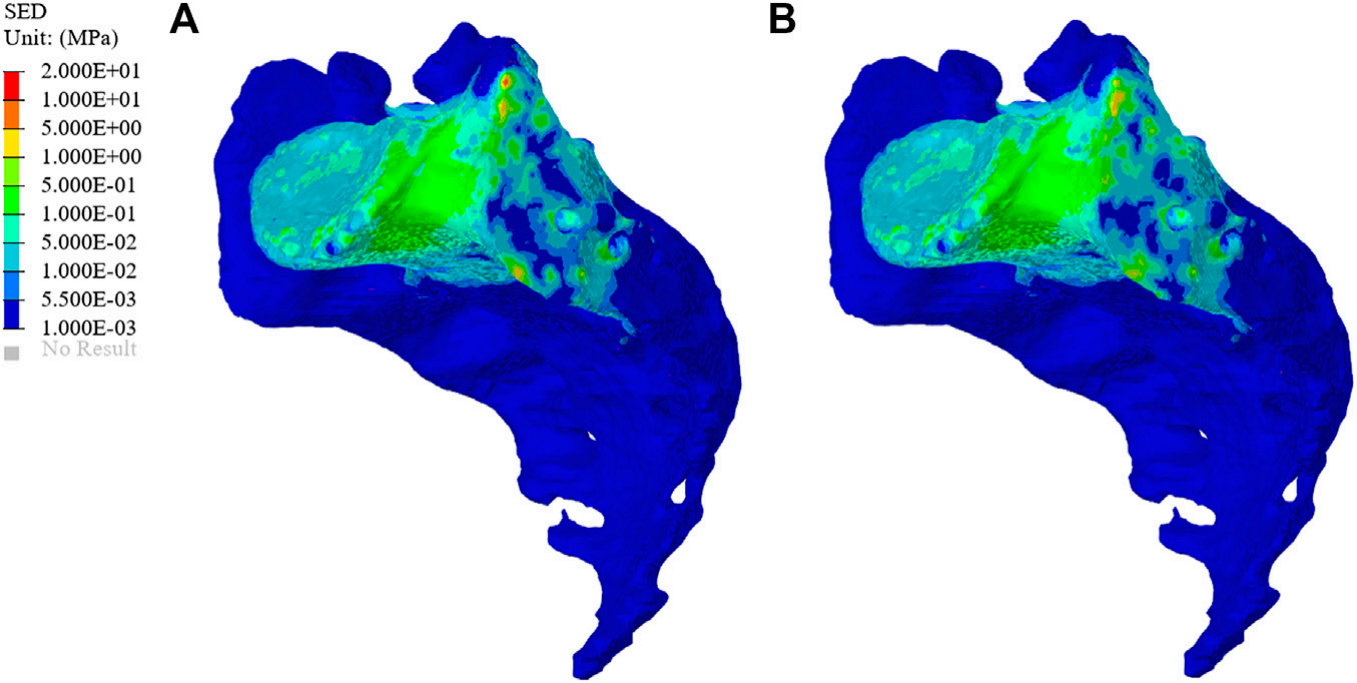
Distribution of sacral strain energy density (SED) in the original group and optimized group. **(A)** The SED distribution of the sacrum in the original group. **(B)** The SED distribution of the sacrum in the optimization group.

The SEDs of the sacrum in the original group and the optimization group are shown in Figure 8. The average SED of sacrum in the original group was 9.96 kPa, and that in the optimization group was 10.80 kPa, showing an increase of 8.43%.

### 3.5 Micromotion Results of Prosthesis

#### 3.5.1 Micromotion Results of Original Prosthesis

**FIGURE 9:**
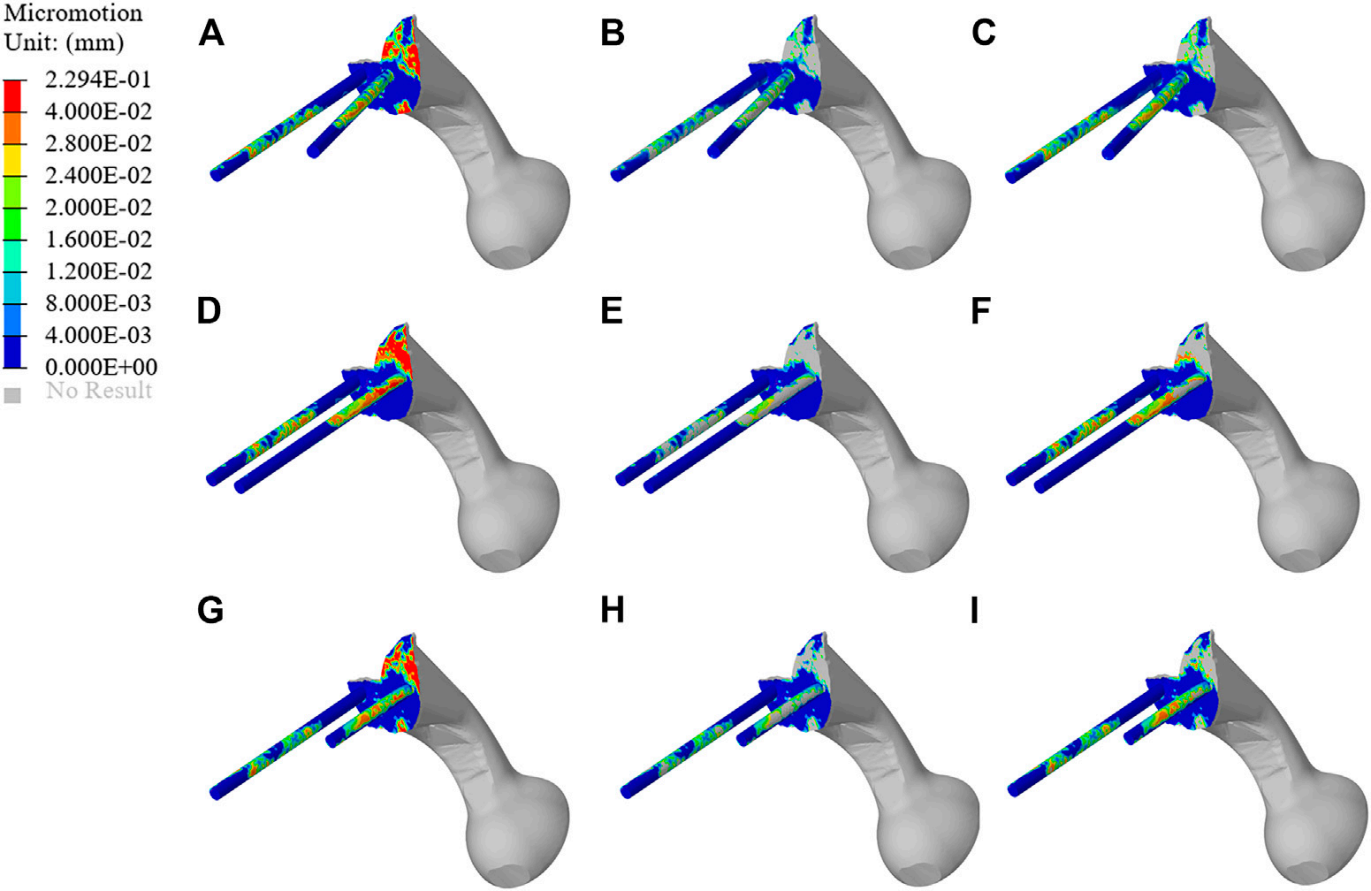
Micromotion results of original prosthesis. **(A–C)** represent the micromotion distribution of BMD screws, **(D–F)** represent the micromotion distribution of long screws, and **(G–I)** represent the micromotion distribution of short screws. The colored parts of **(B,E,H)** represent the area with micromotion less than 28 μm, and the colored parts of **(C,F,I)** represent the area with micromotion less than 40 μm.

The micromotion distribution of prosthesis varies with three different fixation methods. As shown in Figure 9, A, B, and C represent the micromotion distribution of BMD screw, D, E, and F represent the micromotion distribution of long screw, and G, H, and I represent the micromotion distribution of short screw. The colored parts of B, E, and H represent the area with micromotion less than 28 μm, and the colored parts of C, F, and I represent the area with micromotion less than 40 μm. In BMD screw, the area where the micromotion on the screw surface is more than 28 μm is 368.85 mm^2^, and there is no area where the micromotion is more than 40 μm. In long screw, the area of the screw surface with micromotion more than 28 μm is 441.26 mm^2^, and the area of the screw surface with micromotion more than 40 μm is 66.59 mm^2^. In short screw, the area of the screw surface with a micromotion more than 28 μm is 180.93 mm^2^, and the area of the screw surface with a micromotion more than 40 μm is 17.54 mm^2^. The maximum micromotion of the three screws is at the top of the connecting plate. The maximum micromotion of BMD screw is 229 μm, the maximum micromotion of long screw is 191 μm, and the maximum micromotion of short screw is 173 μm.

#### 3.5.2 Micromotion Results of TO Prosthesis

**FIGURE 10:**
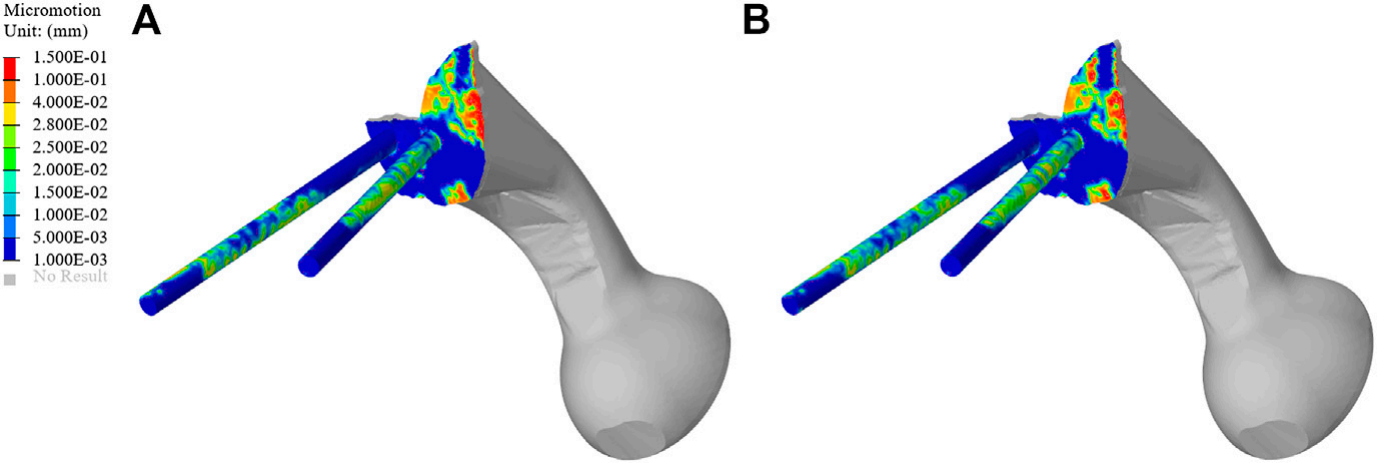
Micromotion results of TO prosthesis. **(A)** Original prosthesis, **(B)** TO prosthesis.

As shown in Figure 10, the micromotion distribution of the original group and the optimization group. A represents the original prosthesis and B represents the topology optimized prosthesis. On the surface of the screw and connecting plate, compared with the original group, the area with micromotion more than 28 μm in the optimization group was reduced from 684.45 mm^2^ to 601.13 mm^2^, a decrease of 12.17%. On the screw surface, area with micromotion more than 28 μm in the optimization group was reduced from 368.85 to 284.38 mm^2^, a decrease of 22.9%. However, the maximum micromotion increased from 229 to 252 μm. Figure 7B shows the average micromotion on S1 screw, S2 screw and connecting plate. The surface micronmotion of each screw was significantly less than that of the original group (*p* < 0.05), and the micromotion on the connecting plate was increased compared with that of the original group.

## 4 Discussion

As one of the most commonly used options for pelvic ring reconstruction, a hemipelvic prosthesis is usually a solid structure with high stiffness, which leads to the non-physiological distribution of stress, and is not suitable for bone integration (Young et al., 2021). The most serious problem caused by this change is stress shielding. Over time, stress shielding leads to bone resorption, facilitating aseptic loosening and even possible fractures around the prosthesis (Kurcz et al., 2018). In addition, the full solid structure without osseointegration does not support long-term stability (Jing et al., 2016). In this study, a new screw channel was determined according to the distribution of sacral BMD, and the screw and connecting plate were subsequently improved based on TO technology and a porous structure so as to enhance biomechanical properties and the stability of the prosthesis. Then, its stability was verified by biomechanics evaluation through FEA.

In order to reduce the stress-shielding effect caused by the mismatch between vertebral cancellous BMD and screw stiffness, this study divided the sacral sagittal plane into several regions and measured the average BMD of each so as to obtain a screw channel with high BMD distribution. The measurement results of sacral BMD clearly showed the distribution pattern of sacral BMD. The BMD in the sacral vertebral body was higher than that in other areas, and the anterior and upper sides of vertebral body was the area with the most highly concentrated BMD. The BMD near the auricular surface was also high, and the posterolateral area was an area with concentrated BMD. Meanwhile, in the sacral wing, the BMD was low. Overall, there were significant differences in BMD distribution at the S1 level, consistent with the results reported by Darius et al. (Thiesen et al., 2020).Lu et al. (2000) previously showed that BMD is a good indicator of the strength of preoperative sacral screw fixation in previous *in vitro* mechanical experiments. Their study showed that the BMD screw can significantly improve the mechanical distribution on the screw, reduce the peak stress on the screw, and decrease the incidence of postoperative complications, consistent with our expected results. Compared to the other two known fixation methods, the area of high-stress concentration at the connection between the screw and the connecting plate can also be eliminated. The position of the BMD screw is similar to the mechanical conduction path of the pelvis (Snijders et al., 1993), so it can render the amount of stress distribution more reasonable. BMD screw effectively reduces the risk of screw loosening or even breaking. The direction of the newly proposed screw channel is the posterolateral entrance of the auricular surface, ending at the contralateral sacral cape. The above results show that this new BMD screw channel is safer than traditional transverse screw fixation and has a lower incidence of complications (Table 3 and 4).

In this study, an inhomogeneous sacral model was reconstructed to simulate the real sacrum. The material properties of the sacrum were determined according to the gray values of CT images. The inhomogeneous sacral model used in this study can improve the accuracy of FEA. Ahmet et al. previously explored the correlation between bone inhomogeneity and reported that inhomogeneous bones can lead to significant differences in stress values compared to rigid bones (Ün and Çalık, 2016). We designed three screw-fixation methods—BMD screw, long screw, and short screw—and by comparing the results of FEA and sacral BMD distribution, we found that the BMD screw had a more reasonable stress distribution. Then, we carried out TO and porous structure surface design. During FEA, von Mises stress can effectively reflect the biomechanical characteristics and record the stress distribution on the screws (Maslov et al., 2021). As shown in Figure 6, the maximum stress of the optimized prosthesis was reduced in this study by 24.39%. The maximum stress of the screw (264.87 MPa) was also lower than the yield strength (789–1,013 MPa) of Ti6Al4V material (Dong et al., 2018), which indicated that the strength of the screws can withstand a static load. In addition, long-term survival of the screw also requires its maximum stress to be far lower than the fatigue strength limit of the material (310–610 MPa) (Long and Rack, 1998). The maximum sacral stress in the optimization group was 133.43 MPa, which was 27.23% lower than that in the original group, without exceeding the yield strength (150 MPa) (Li et al., 2007). At the same time, it was observed that the sacral stress distribution was more uniform and could accept stress stimulation, providing good conditions for bone growth. The results of TO show that TO and a porous structure design can decrease the amount of bone resorption caused by stress shielding so as to reduce the possibility of postoperative prosthesis instability.

Prosthesis micromotion is an important assessment related to prosthesis stability and postoperative pain. Previous studies indicated that 28 μm was considered to be the micromotion boundary value most conducive to bone growth, and the interface micromotion of about 40 μm could lead to partial inward growth, while 100–150 μm was considered to prevent inward bone growth, more than 150 μm would completely inhibit the inward growth of bone (Pilliar et al., 1986; Zhao X. et al., 2018; Zhang et al., 2020). As shown in Figure 9, both BMD screw and long screw can obtain better micromotion results, and have better distal fixation than short screw. However, previous studies have pointed out that the fixation method of long screw may lead to iatrogenic nerve injury and vascular injury (Zhao Y. et al., 2018; Alkhateeb et al., 2020). Therefore, we believe that BMD screw can obtain a larger safe area, meet the requirements of bone growth and obtain satisfactory bone integration. At the same time, As shown in Figure 10, after the topology optimization of the prosthesis, the area of the surface of the screw and connecting plate that is not suitable for bone growth is decreased. This indicated that the BMD screw and prosthesis after topology optimization can achieve satisfactory long-term bone integration effect and reduce patient pain on the basis of meeting the initial stability.

SED is another index for evaluating stress shielding. The difference between the optimized group and the original group can be used to evaluate bone resorption. As shown in Figure 8, the average SED of sacrum in the original group and the optimization group were 9.96 and 10.80 kPa, respectively. The above results show that the combination of TO design and a porous structure plays an important role in realizing the osseointegration of a prosthesis. The surface of a porous structure can reduce the elastic modulus of the prosthesis, and with an increase in porosity, the pore volume of bone growth also increases so as to improve the performance of bone integration (Kelly et al., 2021). Shah et al. (2016) also confirmed that metal implants with an interconnected pore structure have the potential to promote bone growth, and they may also reduce the stiffness mismatch between implant and bone so as to eliminate the stress-shielding effect.

In this new study of prosthesis fixation, we preliminarily verified the initial stability of screw and prosthesis by finite element analysis, and obtained satisfactory results. However, there are some limitations in this study. First, we did not consider the effect of postoperative stress-dependent BMD change on prosthesis fixation. This study exists in our next plan. We will study this phenomenon through animal experiments and postoperative follow-up of patients. Second, we only considered the mechanical interaction between bone tissue and a prosthesis and did not introduce the supporting effect of muscle and ligament on bone or the prosthesis, although the FEA results support the effectiveness in reducing stress shielding. Such operation simplified the finite element model and may cause some deviation in the results. In the future research, we will use a more accurate and more physiological model. Finally, this study only includes a single hemipelvic pelvic model, which may have an impact on the applicability of the prosthesis. Future research will further improve upon the content of this study.

## 5 Conclusion

In this study, we proposed an improved scheme to reduce the probability of stress shielding after pelvic reconstruction, determine a new screw channel, and optimize the structure and porous surface design of the prosthesis. We also used FEA to verify the biomechanical properties of the prosthesis. The maximum stress of the optimized prosthesis was 24.39% lower than that of the original prosthesis, the maximum stress of the sacrum in the optimization group decreased by 27.23%, and the average SED of the sacrum in the optimization group rose by 8.43%. On the surface of screw and connecting plate, the area with micromotion more than 28 μm was reduced by 12.17%. On the screw surface, the area with micromotion more than 28 μm was reduced by 22.9%. The newly determined screw channel and optimized prosthesis design can effectively improve the biomechanical properties of the relationship between a prosthesis and bone. Some limitations of this study, such as the impact of postoperative stress-dependent BMD change on the stability of prosthesis and the failure to use complete pelvis, muscle and ligament structures, may affect the accuracy of the experiment. These limitations have been included in our next experimental plan. This series of studies will provide reference for the stable fixation of prosthesis in clinical pelvic reconstruction.

## Data Availability

The original contributions presented in the study are included in the article/Supplementary Material, further inquiries can be directed to the corresponding author.
